# Cyclic regulation of transcription factor C/EBP beta in human endometrium

**DOI:** 10.1186/1477-7827-7-15

**Published:** 2009-02-17

**Authors:** Beth J Plante, Athilakshmi Kannan, Milan K Bagchi, Lingwen Yuan, Steven L Young

**Affiliations:** 1 Department of Obstetrics and Gynecology, Division of Reproductive Endocrinology and Infertility, University of North Carolina School of Medicine, CB 7570, Old Clinic Building, Chapel Hill, NC 27599, USA; 2 Department of Molecular & Integrative Physiology, University of Illinois at Urbana-Champaign, Urbana, IL, USA

## Abstract

**Background:**

The transcription factor CCAAT/enhancer-binding protein (C/EBP) beta is a critical mediator of murine endometrial function during embryo implantation. Our objective is to characterize changes in C/EBP beta mRNA abundance and protein localization over the normal human menstrual cycle.

**Methods:**

Fifty normally cycling volunteers without reproductive disorders were randomized to undergo endometrial sampling on a specific cycle day, with secretory phase samples timed using urinary LH surge. Samples were assessed for relative C/EBP beta mRNA expression using quantitative real-time RT-PCR and for C/EBP beta protein localization using immunohistochemistry. The semiquantitative histologic scoring (HSCORE) system was used to compare staining intensity in each tissue compartment between each cycle phase.

**Results:**

C/EBP beta mRNA expression by whole endometrium peaks in the late secretory phase and is significantly higher than that in the proliferative and mid-secretory phases. A marked increase in nuclear C/EBP beta protein immunostaining is seen in stromal cells beginning about cycle day 20, coincident with the start of endometrial receptivity. This increased staining continues for the remainder of the cycle.

**Conclusion:**

In the normal human menstrual cycle, C/EBP beta mRNA and protein expression also change, with increased nuclear immunostaining in the mid-secretory phase, suggesting a possible role for C/EBP beta in human endometrial receptivity.

## Background

The transcription factor CCAAT/enhancer-binding protein (C/EBPβ) is a basic leucine zipper (bZIP) family transcription factor [1]. bZIP proteins are involved in the regulation of proliferation, differentiation, metabolic homeostasis, acute phase inflammation, and apoptosis [1-3]. C/EBPβ, in particular, is known to regulate proliferation and differentiation in many target tissues, including liver, adipose tissue, immune cells, skin cells, the ovary, and mammary glands. Depending on the tissue type, C/EBPβ has been shown to either stimulate or inhibit cellular proliferation [1,4-7].

C/EBPβ is a critical mediator of murine endometrial function during embryo implantation. Murine C/EBPβ expression rises rapidly in endometrial epithelium at the time of blastocyst attachment and increases in the endometrial stromal cells around the conceptus during decidualization [8]. Female mice lacking the C/EBPβ gene are infertile due to implantation failure and the uteri of these mice fail to decidualize in response to a mechanical stimulus. Administration of estrogen or progesterone to ovariectomized mice induces C/EBPβ expression in both uterine epithelium and stroma, suggesting that both hormones are involved in the regulation of this gene [8].

While the mouse model suggests that C/EBPβ is regulated by sex steroids and plays an essential role in regulating endometrial function, relatively little is known about C/EBPβ expression and function in human endometrium. Previous studies have demonstrated the presence of C/EBPβ mRNA in human endometrium and upregulation of C/EBPβ during *in vitro *decidualization of endometrial stromal cells [9-12]. Previous studies on *in vivo *regulation of C/EBPβ expression by human endometrium have been limited to small sample sizes and have not allowed specific assessment of the embryo-receptive (mid-secretory) phase of the cycle [11]. Furthermore, no previous work has specifically examined changes in C/EBPβ mRNA abundance.

The purpose of our work is to further define the role of C/EBPβ in human endometrium by characterizing changes in C/EBPβ mRNA abundance, as well as the cellular and subcellular localization of C/EBPβ protein over each functional phase of the normal human menstrual cycle.

## Methods

### Human subjects

50 normal volunteer subjects (ages 18–35, mean age 27 years) with regular cycles (ranging from 25 to 35 days) were randomized to undergo endometrial sampling on a specific cycle day under a protocol approved by the Institutional Review Board at the University of North Carolina at Chapel Hill. These volunteers had no anatomic or functional abnormalities of the reproductive tract and were not taking any medications known to affect reproductive hormone production or action. Proliferative phase samples were timed based on the patient's cycle day, and luteal phase samples were timed using the subject's urinary luteinizing hormone (LH) surge.

Each biopsy was separated into aliquots, with a fraction reserved for RNA analysis and a fraction reserved for fixation in formalin. The portions designated for RNA analysis were flash frozen in liquid nitrogen. The formalin-fixed portions were embedded in paraffin, and sectioned for both immunohistochemistry and staining with hematoxylin and eosin (H&E). Endometrial histology of the H&E sections were evaluated by a single observer (SLY), who was blinded to cycle day and confirmed LH dating in all cases. Samples were categorized as either proliferative (n = 23), early secretory (n = 6), mid-secretory (n = 15), or late secretory (n = 6). All 50 samples were used for quantitative real-time reverse transcriptase-polymerase chain reaction (RT-PCR). Eleven of these samples were used for immunohistochemistry, in addition to nine previously collected samples. The 20 samples used for immunohistochemistry were evenly distributed throughout the menstrual cycle. One slide was of poor quality and was not suitable for H-scoring. Two additional slides did not exhibit any luminal tissue and were, therefore, excluded from scoring for that particular compartment.

### RNA isolation and quantification

Quantitative real-time RT-PCR was performed on total RNA, in triplicate, using probe-primer sets specific for C/EBPβ and the constitutively-expressed gene, cyclophilin (PPIA) (Gene Expression Assays, Applied Biosystems – assay ID HS00270923_s1 (C/EBPβ) and HS99999904_m1 (PPIA)). These probe-primer sets cross introns, and therefore, are expected to provide signal only from mRNA and not from genomic DNA. Cyclophilin was chosen because previous work suggested that cyclophilin exhibits little variation across the menstrual cycle (SLY unpublished data). In the current experiment, Ct values were consistent between samples, further confirming the constitutive expression of this gene.

Endometrial total RNA was isolated from frozen tissue samples using the RNAqueous-4 PCR Kit (Ambion) according to the manufacturer's suggested conditions. RNA quantification was performed using RiboGreen (Invitrogen) with a ribosomal RNA standard curve. First strand complementary DNA (cDNA) was synthesized from 500 ng of total RNA (Roche 1^st ^Strand cDNA Synthesis Kit for RT-PCR). An equivalent volume of water was substituted for the RNA for each reaction as a "no template" negative control. The total reaction volume was 20 μl, and reverse transcription conditions were 25°C for 10 minutes, 42°C for 60 minutes, 99°C for 5 minutes.

Each sample of cDNA was diluted 1:5, and plated in triplicate with TaqMan Master Mix (Applied Biosystems) and sterile water. Primers and probes for C/EBPβ and cyclophilin (PPIA) were obtained in a predesigned mix for each gene. The total reaction volume for all real-time PCR experiments was 20 μl. Reactions were performed in 96-well plates on a Stratagene MX3000 device for 40 2-step cycles (95°C for 25 seconds and then 60°C for 1 minute).

The efficiency of all PCR reactions was 84.1% (C/EBPβ) and 82.3% (PPIA). The average cycle number at which the TaqMan fluorescence became detectable above the threshold (Ct) was 25.9 (C/EBPβ) and 25.4 (PPIA). Ct values were converted to relative expression using the delta-delta Ct method, allowing normalization to both the housekeeping gene, PPIA, and a single sample in the proliferative phase. The RT-PCR data were grouped by cycle phase and analyzed by one way analysis of variance using Tukey's Multiple Comparison Test for post hoc analysis.

### Immunohistochemistry

Paraffin embedded endometrial sections were deparaffinized in xylene, rehydrated in decreasing concentrations of ethanol, and rinsed in phosphate buffered saline for five minutes. Antigen retrieval was performed using 0.1 M citrate buffer at pH 6.0. Endogenous peroxidase activity was blocked by incubating the slides with 0.3% H_2_O_2 _in methanol for 30 minutes. Slides were washed in water followed by incubation with 10% goat serum in PBS for an hour at room temperature to block non-specific binding. The sections were then incubated with a rabbit polyclonal antibody against C/EBPβ (C-19, Santa Cruz Biotechnology, Santa Cruz, CA) diluted 1:200 in blocking solution in a humid chamber overnight at 4°C. The sections were then incubated with goat anti-rabbit biotinylated IgG (Histostain Kit, Zymed Laboratories Inc., San Francisco, CA) for thirty minutes. Finally, horseradish peroxidase (HRP)-conjugated streptavidin was added and incubation was performed for thirty minutes at room temperature. The sections were stained in aminoethylcarbazole (AEC) solution (Zymed Laboratories Inc., San Francisco, CA) for one to three minutes until optimal signals were obtained. In addition, adjacent sections with no primary antibody were used as controls.

Staining intensity and location was measured by two independent observers using the semiquantitative histologic scoring (HSCORE) system. The following equation was used to calculate HSCORES: HSCORE = ΣP_i _(I + 1). I represents the staining intensity (values of 1, 2, or 3 signify weak, moderate, or strong staining, respectively), and P_i _is the percentage of stained cells for each intensity category (ranging from 0–100%). Previous work has demonstrated the low intraobserver and interobserver variability of the HSCORE system applied to immunostaining of endometrium [13]. Disagreements in HSCORE were resolved by consensus conference between the observers. The HSCORES were grouped by cycle phase and analyzed by one way analysis of variance using Tukey's Multiple Comparison Test for post hoc analysis.

## Results

**Figure 1:**
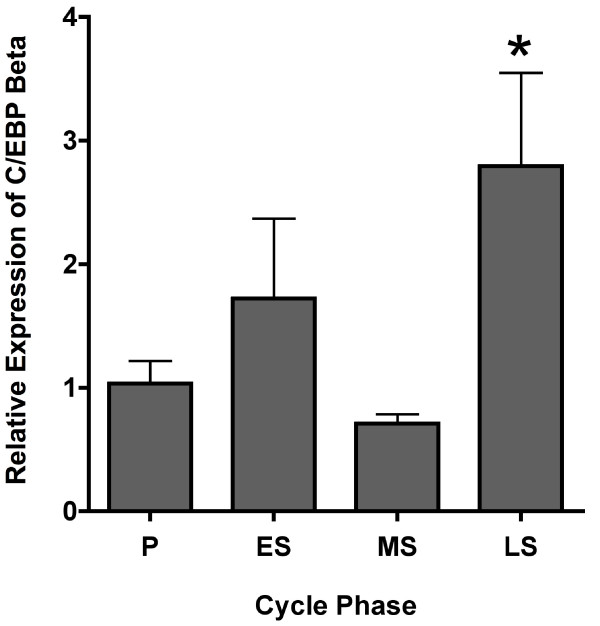
**Endometrial C/EBPβ mRNA expression**. Relative expression of C/EBPβ mRNA by whole endometrium across the menstrual cycle (P = proliferative, ES = early secretory, MS = mid-secretory, LS = late secretory). *C/EBPβ expression in the late secretory phase was significantly higher than that in the proliferative (*P *< 0.01) and mid secretory phases (*P *< 0.001).

Timed endometrial biopsies from healthy volunteers were obtained in the proliferative (n = 23), early secretory (n = 6), mid-secretory (n = 15), or late secretory (n = 6) phase of the menstrual cycle and RNA was extracted. Relative expression of C/EBPβ mRNA by whole endometrium was found to peak in the late secretory phase and was significantly higher than that in the proliferative (*P *< 0.01) and mid-secretory phases (*P *< 0.001) (Figure. 1). The increase in the late secretory phase mRNA was predominantly due to marked increases at 13 and 14 days after the LH surge.

**Figure 2:**
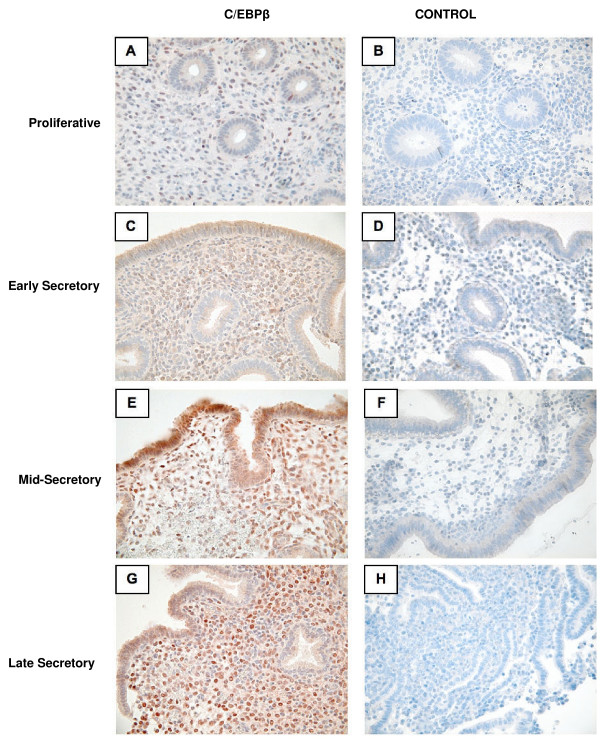
**Immunohistochemical C/EBPβ localization across the menstrual cycle**. Proliferative phase (A, B). Early secretory phase (C, D). Mid-secretory phase (E, F). Late secretory phase (G, H). C/EBPβ staining is shown in A, C, E, and G, while controls for non-specific staining (B, D, F, and H) utilized identical conditions except the primary antibody was omitted.

In order to correlate C/EBPβ mRNA and protein expression, as well as characterize the cellular and subcellular localization of C/EBPβ protein over the normal human menstrual cycle, immunohistochemical analysis was performed (Figure. 2). HSCOREs were analyzed in order to more accurately quantify the immunohistochemical findings. Composite HSCORES of overall endometrial C/EBPβ immunostaining (nuclear plus cytoplasmic staining) revealed no significant change across the menstrual cycle, whether taken as a whole or specifically in stroma, glands, or luminal compartments (not shown).

**Figure 3:**
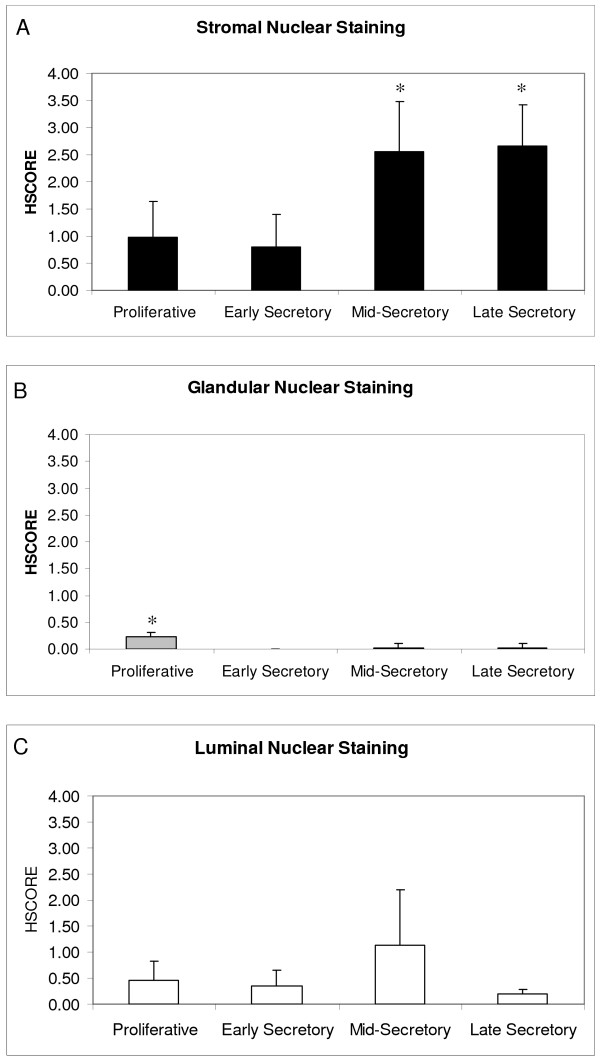
**HSCORES of nuclear C/EBPβ endometrial immunostaining**. HSCORES of nuclear endometrial immunostaining for C/EBPβ in stroma (A), glands (B), and lumen (C) across the menstrual cycle. *HSCORES reveal a significant increase in nuclear stromal C/EBPβ expression in the mid- and late secretory phases.(p < 0.05). Nuclear glandular staining was significantly higher in the proliferative phase compared to the secretory phase (p < 0.01).

C/EBPβ, however, acts specifically in the nucleus, and specific assessment of nuclear staining demonstrated significant changes in the stromal and glandular compartments. Stromal nuclei demonstrated low staining in the proliferative and early secretory phases with significant increases in the mid- and late secretory phases (p < 0.05) (Figure. 2 & Figure 3A).

An increase in glandular cytoplasmic staining was seen during the secretory phase, but nuclear staining showed a significant decrease when compared with the proliferative phase (p < 0.01)(Figure. 3B). While the change in nuclear staining was statistically significant, its physiologic significance is questionable since glandular nuclear staining was very weak across the entire cycle. Modest luminal nuclear staining for C/EBPβ was seen throughout the menstrual cycle, with a non-significant trend towards increasing in the mid-secretory phase (Figure. 2 and Figure 3C).

## Discussion

Murine studies demonstrate that C/EBPβ plays an essential role in mediating the biological activity of estrogen and progesterone in the mouse uterus at the time of embryo implantation [1,8]. C/EBPβ mRNA is also present in human endometrium and is upregulated in vitro during stromal cell decidualization [9-12]. The purpose of our work has been to further define the role of C/EBPβ in human endometrium by characterizing changes in C/EBPβ mRNA abundance, as well as the cellular and subcellular localization of C/EBPβ protein over the normal human menstrual cycle.

Our mRNA results demonstrate peak C/EBPβ mRNA expression in the late secretory phase in whole samples of human endometrium, primarily at LH+13 and LH+14, with no increase seen in the mid-secretory phase. The finding of very late secretory increase suggests a potential role for increased C/EBPβ in preparation for menstruation. The lack of a mid-secretory mRNA increase was surprising, since human stromal decidualization begins during this phase. Additionally, C/EBPβ is induced in human endometrial stromal cells by *in vitro *decidualization, and previous microarray studies of human endometrium showed a small (< 2-fold) increase in the mid-secretory versus early secretory phase and a further small (< 2-fold) increase in the late secretory versus mid-secretory phase [9]. Furthermore, C/EBPβ plays a critical role in murine endometrial decidualization. The apparent discrepancy in our mid-secretory findings versus previous microarray data may be explained, in part, by the relative lack of precision of microarray studies or a change too small to be easily seen by RT-PCR. Alternatively, these differences may simply be due to individual variations within human cycle phases.

In order to shed further light on the mRNA results and clarify the cellular and subcellular localization of C/EBPβ protein, immunohistochemical analysis was performed. A slight increase in C/EBPβ cytoplasmic staining is seen in the glands during the secretory phase. This glandular staining, however, is primarily cytoplasmic and may represent an inactive form of C/EBPβ, because the active, DNA binding, phosphorylated form of C/EBPβ localizes to the nucleus. Depending on the physiological state of the endometrial cells, C/EBPβ may be present in the cytoplasm before being activated by a phosphorylation event and subsequently translocated to the nucleus [14-16].

Nuclear immunostaining increases markedly in the subepithelial stromal cells in the mid-secretory phase. It is of particular interest that the increase in stromal C/EBPβ nuclear staining coincides with the opening of the window of receptivity to embryo implantation. Overall, the human stromal C/EBPβ immunohistochemical findings are consistent with those in the murine model as both systems show a rise in C/EBPβ during the receptive phase [8]. Additionally, beginning in the mid-secretory phase, stromal cells undergo predecidualization. C/EBPβ 's increased stromal nuclear expression may indicate a role for this transcription factor during this differentiation process. This rise in nuclear stromal C/EBPβ staining seen in the human during the mid- and late-secretory phases is consistent with the murine rise during decidualization. Human endometrial stroma undergoes partial decidualization (predecidulaization) in the mid-secretory phase, whereas murine stroma normally decidualizes only during implantation. In both cases, increased nuclear stromal C/EBPβ staining is seen in conjunction with the decidualization process.

When taken together, the mRNA and protein analyses illustrate provocative changes in C/EBPβ expression across the human menstrual cycle. The RT-PCR findings help to accurately quantify relative changes in C/EBPβ mRNA expression in *whole *endometrium throughout the menstrual cycle. These results represent a composite picture of the changes in epithelial and stromal expression of C/EBPβ. Differences between composite endometrial mRNA and immunohistochemical findings may be due to differences in cytoplasmic versus nuclear localization. For instance, a change in overall expression of C/EBPβ may mask a modest but significant enhancement in nuclear expression in a given compartment. Furthermore, C/EBPβ expression has been documented in immune cells where it is important for macrophage cytokine expression and B-lymphocyte function [17]. Additionally, there is a known influx of immune cells, including macrophages, into endometrial stroma prior to menstruation [18]. Because of this relationship, we speculate that the late secretory rise in endometrial C/EBPβ mRNA may be due to this influx of immune cells.

To our knowledge, this paper is the first description of C/EBPβ expression that allows analysis of changes in expression over each functional stage of endometrial differentiation. Previous work demonstrated diffuse cellular localization of C/EBPβ protein in human endometrium [11], but our current findings demonstrate the expected nuclear localization of this transcription factor in both the luminal and stromal compartments during the receptive phase of the menstrual cycle. These discrepancies may be due to differences in the immunohistochemistry protocols that were employed. This previous work was also somewhat limited by relatively few samples of human endometrium (eighteen total). In addition, the three functional phases of secretory endometrium were not analyzed separately, presumably because of the small pool of available samples. Instead, specimens from the secretory phase were simply categorized as either early or late secretory. Such a classification system omits a separate analysis of the mid-secretory samples, which would be the samples of most interest as they should reflect endometrial changes during the window of endometrial receptivity to embryo implantation.

## Conclusion

In summary, C/EBPβ mRNA and protein expression noticeably change throughout the normal human menstrual cycle and particularly during the embryo-receptive phase, suggesting a possible role for C/EBPβ in human endometrial function and embryo implantation. Human stromal C/EBPβ protein changes are largely consistent with those in the murine model. Further studies are needed to elucidate the precise mechanisms by which C/EBPβ influences these biological processes in normal human endometrium. In addition, potential differences in C/EBPβ expression in the endometrium of infertile women remain unknown but warrant investigation.

## Competing interests

The authors declare that they have no competing interests.

## Authors' contributions

BJP assisted with the recruitment of human subjects and endometrial sampling, participated in the RT-PCR, performed manual HSCORES, and drafted the manuscript. AK performed the immunohistochemistry and assisted with experimental design. MKB conceived of the study in conjunction with SLY, participated in its design and coordination, and helped to draft the manuscript. LY carried out the RNA isolation and quantification and provided significant input into experimental method development. SLY conceived of the study in conjunction with MKB, participated in its design and coordination, assisted with the manual HSCORES, helped with the statistical analysis, and helped to draft the manuscript. All authors read and approved the final manuscript.
